# Broca’s aphasia due to cerebral venous sinus thrombosis following chemotherapy for small cell lung cancer: A case report and review of literature

**DOI:** 10.3892/ol.2014.2709

**Published:** 2014-11-19

**Authors:** TOLGA TUNCEL, ALPASLAN OZGUN, LEVENT EMIRZEOĞLU, SERKAN CELİK, SERKAN DEMİR, OGUZ BILGI, BULENT KARAGOZ

**Affiliations:** 1 Department of Medical Oncology, Gata Haydarpasa Training Hospital, Istanbul 34668, Turkey; 2 Department of Neurology, Gata Haydarpasa Training Hospital, Istanbul 34668, Turkey

**Keywords:** Broca’s aphasia, sinus venous thrombosis, cisplatin, lung cancer

## Abstract

Cancer is associated with an increased risk of cerebrovascular incidents and treatment with chemotherapy enhances that risk further. Brocha’s aphasia is a stroke-related syndrome, the presentation of which has been rarely reported during cisplatin-based chemotherapy. The current study presents the case of a 27-year-old male with advanced-stage small cell lung cancer. The patient developed Broca’s aphasia following cisplatin-based chemotherapy.

## Introduction

Broca’s aphasia is a condition resulting from damage to speech areas in the left hemisphere (1). It is easily distinguished by experienced clinicians from other types of aphasia, such as Wernicke’s, global and conduction aphasia; however, the prognosis of patients with Broca’s aphasia remains poor (2). Cerebral venous sinus thrombosis (CVST) is a rare condition, accounting for 0.5–1% of all strokes and can cause Broca’s aphasia (2). It mostly presents as a seizure, intracranial hypertension syndrome, isolated headache and focal lobar syndrome (3). Diagnosis of CVST is usually based on the presence of thrombi in the cerebral sinuses and/or veins on veno computed tomography or magnetic resonance venography (4). Cisplatin is an antineoplastic agent and has been associated with cerebrovascular incidents (5). Although the exact mechanism of cisplatin-induced thromboembolic events is not currently well understood, direct vascular toxicity, apoptosis, endothelial dysfunction, hypomagnesemia and tumour embolisation are possible risk factors (6). The current study presents the case of a young male patient with advanced-stage small cell lung cancer who developed Broca’s aphasia following cisplatin-based chemotherapy, and also reviews the literature surrounding similar cases. Written informed consent was obtained from the patient.

## Case report

**Figure 1:**
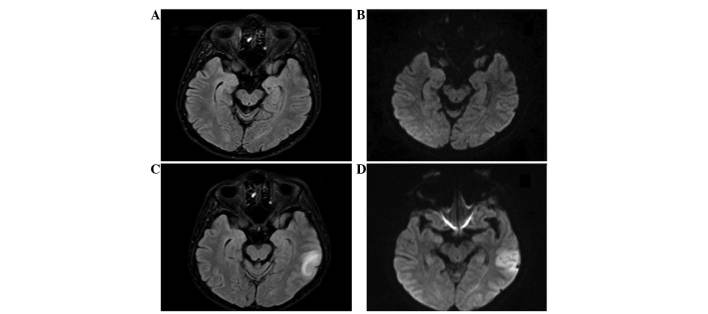
(A and B) Normal cranial magnetic resonance imaging and (C and D) flair and diffusion weighted images showing acute left parietal infarction.

**Figure 2:**
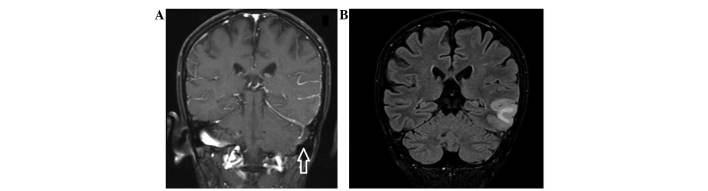
(A) Contrast enhanced coronal T1 weighted imaging shows left transverse sinus thrombosis and (B) flair coronal sequence shows infarction.

A 27-year old male presented to the Department of Medical Oncology, Gata Haydarpasa Training Hospital (Istanbul, Turkey) with Broca’s aphasia and general seizures. The patient had been diagnosed with advanced-stage small cell lung cancer two months previously. Baseline magnetic resonance imaging (MRI) of the patient was normal (Fig. 1). The patient received 135 mg cisplatin on day one and 180 mg etoposide on days one to three. On day six of the second cycle, the patient was admitted to the emergency department 4 h following the onset of a stroke. The MRI scan revealed a CVST in the left transverse sinus, as well as in the left sigmoid sinus with venous infarct (Fig. 2). Laboratory analysis showed a normal coagulation profile (activated partial thromboplastin time, 34.5 sec; normal range, 25–38 sec; international normalized ratio, 1.048; normal range, 0.8–1.2), and serum magnesium levels (2 mg/dl; normal range, 1.2–2.5 mg/dl), platelet count (204,000/mm^3^; normal range, 150,000–450,000/mm^3^) and lipid profiles (triglycerides: 116 mg/dl; normal range, 40–160 mg/dl) were also normal. Echocardiography for embolic sources and electrocardiography for arrhythmia were normal. The patient was subjected to anticoagulation therapy with low molecular weight heparin and speech therapy was commenced. The symptoms resolved completely within one month and cisplatin-etoposide chemotherapy was continued in addition to the anticoagulation therapy.

## Discussion

Chemotherapy leads to an increased risk of thromboembolic complications in germ cell, urethral, head and neck and breast cancer, lymphoma and leukemia (7,8). Venous thromboembolic events are observed more frequently than arterial thromboembolic events. Weijl *et al* (7) reported that germ cell cancer patients who receive cisplatin-based chemotherapy, particulary those who receive high doses of corticosteroids or have liver metastases, are at considerable risk of developing thromboembolic events. Prophylactic administration of heparin may be considered in this group. Li *et al* (8) reviewed 10,963 patients and reported that the risk of ischemic stroke stroke after chemotherapy is predicted by the use of cisplatin-based chemotherapy, not cancer histological type. The worldwide incidence of venous thromboembolic events during cisplatin-based chemotherapy for a various advanced solid tumors is 1.92% (10). We reviewed the literature with respect to CVST occurring in cisplatin-based chemotherapy. To date, very few cases of patients with CVST following cisplatin-based chemotherapy have been reported (Table I) (11–13) and to the best of our knowledge, this is the first report of Broca’s aphasia and CVST associated with a cisplatin-based chemotherapy regimen.

The patient in the current study presented with Broca’s aphasia due to a CVST in the left transverse sinus, following cisplatin-based chemotherapy. Increased von Willebrand factor, hypomagnesemia and damage to the vascular endothelium are known risk factors for the cisplatin-associated vascular toxicity. Chemotherapy-induced venous thrombosis usually develops within 10 days following the most recent chemotherapy cycle and 63% of cases occurred following the first cycle (9). CVST is rare condition responsible for approximately 1–2% of all cerebral strokes (14). The clinical presentation of CVST is highly variable and non-specific; symptoms including headaches, nausea and vomiting, visual disturbances, aphasia, coma and seizure may be observed in CVST. The treatment of this condition ranges from anticoagulation with intravenous heparin or subcutaneous low-molecular-weight heparin, to endovascular thrombectomy or thrombolysis. To date, there are no evidence-based guidelines on prophylaxis of thromboembolic events in cancer patients that are treated with cisplatin-based chemoterapy. Prophylactic heparin may be used in patients with curable germ cell tumors and early stage of lung cancer. The aim of this study was to increase the awareness of the possibility of CVST in patients with lung cancer treated with cisplatin-based chemotherapy.

In conclusion, the possibility of Broca’s aphasia and CVST in patients with lung cancer, receiving platinum-based chemotherapy, must be considered when determining the differential diagnosis. Broca’s aphasia is a fatal and rare complication, but can be treated successfully with low-molecular-weight heparin. An accurate diagnosis of CVST may be determined using MRI. To date, there are no evidence-based guidelines for the prophylaxis of thromboemboli in cancer; for patients with a history of thromboembolism, the concomitant use of low-molecular-weight heparin and acetylsalicylic acid during each cycle of cisplatin-based chemotherapy must be considered (15).

## Figures and Tables

**Table I:** Previously reported cases of cerebral venous sinus thrombosis following cisplatin-based chemotherapy.

First author (year) [ref]	Age, years/gender	Tumor	Chemotherapy	Symptoms	Imaging	Site	Treatment
Unal (2008) [10]	16/F	Ewing sarcoma	Cis+Ifo+Adr+Vin	Headache diplopia ptosis	MR MRA	SSSTS	LMWH
Karam (2008) [11]	33/M	Germ-cell carcinoma of the testis	Cis+Eto	Headache, partial seizures	MR, CA	SSS RLS	Anticoagulant sodium valproate
Karam (2008) [9]	60/F	PDP carcinoma	Cis+5 Fluorouracil	Headache, partial seizures	MR, CA	SSS RLS	Heparin, clopidogrel sodium valproate
Papet (2011) [12]	47/M	Embryonal carcinoma	Cis+Eto+Ble	Headache, weakness in left limbs	MR	SSS SS TS	Anticoagulant
Papet (2011) [12]	29/M	Embryonal carcinoma	Cis+Eto+Ble	Headache, general seizure	CT	SSS	LMWH

F, female; Cis, cisplatin; Ifo, ifosfomid; Adr, adriamycine; MR, magnetic resonance; MRA, magnetic resonance angiography; SSS, superior sagittal sinus; TS, transverse sinus; LMWH, low molecular weight heparin; M, male; Eto, etoposide; RLS, right lateral sinus; CA, conventional angiography; PDP, poorly differentiated pericardial carcinoma; Ble, bleomycin; SS, sigmoid sinus.
